# Association of Pregnancy With Recurrence of Spontaneous Coronary Artery Dissection Among Women With Prior Coronary Artery Dissection

**DOI:** 10.1001/jamanetworkopen.2020.18170

**Published:** 2020-09-23

**Authors:** Marysia S. Tweet, Kathleen A. Young, Patricia J. M. Best, Meredith Hyun, Rajiv Gulati, Carl H. Rose, Sharonne N. Hayes

**Affiliations:** 1 Department of Cardiovascular Diseases, Mayo Clinic College of Medicine and Science, Rochester, Minnesota; 2 Department of Biomedical Statistics and Informatics, Mayo Clinic College of Medicine and Science, Rochester, Minnesota; 3 Division of Maternal and Fetal Medicine, Department of Obstetrics and Gynecology, Mayo Clinic College of Medicine and Science, Rochester, Minnesota

## Abstract

**Importance:**

Spontaneous coronary artery dissection (SCAD) is a notable cause of acute coronary syndrome in women of childbearing age.

**Objective:**

To test the hypothesis that pregnancy after SCAD is associated with recurrent SCAD.

**Design, Setting, and Participants:**

Three study designs were implemented: a case series of women with pregnancy after SCAD; a nested case-control study comparing patients with recurrent SCAD to matched controls without recurrent SCAD; and a cohort study. Women with SCAD who were of childbearing potential and enrolled into the Mayo Clinic SCAD Registry from August 30, 2011, to April 4, 2019, were included in the study. Patients with coronary dissections associated with iatrogenesis, trauma, or atherosclerosis were not enrolled.

**Exposures:**

Pregnancy after SCAD.

**Main Outcomes and Measures:**

The primary outcome was SCAD recurrence, defined as an acute coronary syndrome or cardiac arrest due to new SCAD. Other demographic measures collected included age, year of SCAD occurrence, and comorbidities.

**Results:**

The cohort included 636 women of childbearing potential. Twenty-three of those women had a total of 32 pregnancies after SCAD. The median (interquartile range) age of women with pregnancy after SCAD was 38 years (34-40 years), and 20 (87%) were White. In the nested case-control study, 92 cases of recurrent SCAD were matched to 158 controls. There was no significant difference in exposure to subsequent pregnancies in the women with recurrent SCAD as compared with matched controls (2 of 92 [2%] vs 13 of 158 [8%]; *P* = .06). In the overall cohort of 636 patients, recurrent SCAD was present in 122 patients with a Kaplan-Meier 5-year SCAD recurrence estimate of 14.8%. The Cox analysis showed no significant association between subsequent pregnancy and SCAD recurrence with a nonsignificant hazard ratio of 0.38 (95% CI, 0.09-1.6) when controlling for age at first SCAD, year of first SCAD, and fibromuscular dysplasia.

**Conclusions and Relevance:**

This study found that most women tolerated pregnancy and lactation after SCAD without evidence for increased risk of SCAD recurrence when compared with women with a history of SCAD who did not experience pregnancy. Although this study is reassuring and indicates complex contributors to SCAD recurrence, the results need to be interpreted prudently because of study selection bias and the small total number of women who became pregnant after SCAD. The notable hemodynamic changes that occur with pregnancy and severe presentation of pregnancy-associated SCAD are reasons for concern when considering pregnancy after SCAD.

## Key Points

**Question:**

What is the risk of recurrent spontaneous coronary artery dissection (SCAD) with pregnancy in women with a history of coronary artery dissection?

**Findings:**

In this nested case-control study of 636 women, there was no difference in exposure to pregnancy after SCAD among women with recurrent SCAD as compared with matched women without recurrent SCAD. There was no association between subsequent pregnancy and recurrent SCAD at 5 years’ follow-up in the overall cohort.

**Meaning:**

This study found no evidence for increased risk of SCAD recurrence among women who became pregnant after SCAD, but results are limited by the small total number of women with normal left ventricular ejection fraction at time of subsequent pregnancy and should be interpreted with caution.

## Teaser

This nested case-control study uses data from the Mayo Clinic Spontaneous Coronary Artery Dissection Registry to investigate the association of pregnancy with spontaneous coronary artery dissection recurrence among women with a history of coronary artery dissection.

## Introduction

Spontaneous coronary artery dissection (SCAD) is a nonatherosclerotic cause of acute coronary syndrome that occurs in women of childbearing age.^1,2^ It is the cause of ST-elevation myocardial infarction (MI) in 1 of 5 women younger than age 50 years^3^ and a foremost cause of heart attack among pregnant women.^1,4,5^ Acute SCAD causes MI or sudden cardiac death due to obstruction by a coronary artery wall hematoma alone or a hematoma and an intimal-medial tear.^1,6^ Enhanced awareness and technological improvements in the catheterization laboratory have facilitated the diagnosis of SCAD,^1^ which for some patients has been previously overlooked.

More than 80% of patients with SCAD are women with a mean age of 42 to 52 years at the time of SCAD occurrence.^1,2^ Most patients with a history of SCAD do not have the traditional risk factors, such as tobacco use, hyperlipidemia, diabetes, or a sedentary lifestyle.^7^ Approximately one-third of patients have a history of hypertension.^8,9^ Most patients have abnormalities in the peripheral arteries, such as fibromuscular dysplasia (FMD), aneurysms, and dissections.^1,10,11,12,13^ Only a small proportion of patients have an inherited connective tissue disease (5%-8%)^14,15^ or family members with SCAD (1%).^16,17,18^ Despite the observation based on follow-up imaging that nearly all coronary dissections heal, the morbidity of SCAD is substantial, with an estimated 30-day rate of major adverse cardiac events of 8.8%^8^ and 10-year estimated rate of major adverse cardiac events as high as 47%.^6^ Much of the long-term risk is related to SCAD recurrence, which occurs in 12% to 29% of patients.^6,19^ SCAD recurrence is defined as an MI or cardiac arrest due to SCAD in another, usually different, coronary artery.^1^ Risk factors for SCAD recurrence and effective prevention strategies are unknown and remain a principal concern for patients and clinicians.^19,20^

The onset of SCAD symptoms has been associated with the pregnant and postpartum condition, exertion, and emotional or psychological stress.^1,2,21,22^ The female sex hormonal milieu likely contributes to its pathophysiology as evidenced by the marked female preponderance, association with pregnancy, and post-SCAD chest pain associated with menses.^1,2,22^ Subsequent reproductive decisions are challenging because of concern for adverse events, and the current expert consensus is to discourage pregnancy after SCAD.^1^ We aimed to describe short- and long-term outcomes in women with post-SCAD pregnancy to better inform future counseling of childbearing women after SCAD.

## Methods

### Study Population

In response to patient advocacy for more research about SCAD in 2009, the Mayo Clinic initiated an ongoing international registry and biorepository of patients with SCAD,^23,24^ which were used for this nested case-control study. This work was approved by the Mayo Clinic Institutional Review Board. We followed the Strengthening the Reporting of Observational Studies in Epidemiology (STROBE) reporting guideline and checklist for case-control studies. Patients provided written informed consent and were included in the registry after review and confirmation of SCAD on coronary angiogram imaging by at least 2 interventional cardiologists. Patients with coronary dissections associated with iatrogenesis, trauma, or atherosclerosis were not enrolled. Data collection consisted of exhaustive questionnaires, medical history, imaging, and prospective follow-up. From the time of the pilot study in 2010^25^ to ongoing, over 1200 participants with confirmed SCAD from throughout the United States (96%) and other countries (4%) have been enrolled.

For the present study, all women who consented into the Mayo Clinic SCAD Registry from August 30, 2011, through April 4, 2019, were reviewed for a history of pregnancy after SCAD. This was conducted by reviewing responses to specific questions about pregnancy after SCAD on the initial study entry questionnaire and 2 follow-up surveys. Additionally, the clinical records of participants who are concurrent patients of the Mayo Clinic SCAD Clinic were reviewed for a post-SCAD pregnancy. Time to follow-up for all patients was determined by date of last contact by survey, phone, email, or clinical evaluation. To test the hypothesis that recurrent SCAD is associated with pregnancy after SCAD, we conducted cohort, case series, and nested case-control analyses (Figure).

**Figure.**
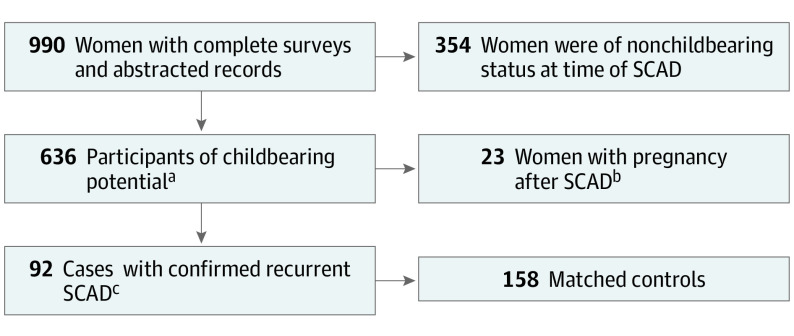
Study Participants Identification of participants for the 3 analyses as described in the methodology. SCAD indicates spontaneous coronary artery dissection. ^a^Cohort analysis. ^b^Case series analysis. ^c^Case-control analysis.

### Case Series of Women With Post-SCAD Pregnancies

Given the paucity of information regarding women with post-SCAD pregnancies, extensive details of pregnancy outcomes, complications, and current reproductive status were collected by email, phone call, or clinical follow-up. Twenty-three women were identified as having a total of 32 post-SCAD pregnancies. Although 15 have been evaluated in the Mayo Clinic SCAD Clinic, only 2 were seen in clinical follow-up during the course of the present study. The other 21 were contacted by phone or email for additional details and follow-up information, including 8 women who were previously described.^26^

### Nested Case-Control

To leverage the registry, a nested case-control study was also performed. The demographic characteristics of all women with SCAD in the registry cohort with complete records were reviewed. Those who were not of childbearing age at the time of initial SCAD were excluded. This was determined by identifying women who marked a reproductive status of hysterectomy, menopause, or postmenopause at time of initial SCAD and women who were older than age 56 years at the time of SCAD. As recurrence is a primary endpoint of interest from both a mechanistic and clinical perspective, cases were identified as patients who had a recurrent SCAD. Recurrent SCAD was defined as an MI or cardiac arrest distinct from the initial SCAD based on detailed physician review of surveys, medical records, and imaging. Because the American Heart Association SCAD consensus statement indicates that SCAD recurrence within 1 month of the initial event could represent continuation of the initial event,^1^ SCAD recurrence for the purposes of case identification was limited to those who had recurrence at least 1 month after the initial SCAD. Patients who experienced initial SCAD progression (distinct from recurrence at <1 month), MI from other causes, or other complications, such as in-stent restenosis, were not categorized as having recurrent SCAD. After the cases were identified, controls were matched to each case in an approximate 2:1 ratio. The matched factors were FMD status, age at first SCAD, and year of first SCAD. Exact matching of FMD status was required. Age at first SCAD and year of first SCAD were matched as closely as possible but allowed to vary as much as 8 and 9 years, respectively. FMD status (categories: not imaged or unknown, yes, non-FMD arteriopathy, no, and possible) was included because of concern that this could be a confounder for recurrence. Matching on age was prioritized over year of first SCAD to allow for similar childbearing potential between the groups. The algorithm also required controls to have been followed at least as long as the cases they were matched to, ensuring opportunity for recurrence would be the same within each match. Matching according to year of first SCAD was to account for changes in SCAD recognition and management because of recent advancements in knowledge about SCAD. Once established, cases and controls were compared on numerous variables of interest, including post-SCAD pregnancy occurrence.

### Cohort Time to Event

Because of concern regarding the small number of women with pregnancy after SCAD and that some would be excluded after the matching process for the nested case-control study, a cohort time-to-event analysis was also performed with the 636 participants identified as being of childbearing age at time of initial SCAD. The analysis was performed twice using 2 definitions of SCAD recurrence (any time after SCAD vs ≥1 month after SCAD).

### Statistical Analysis

Statistical analysis was conducted using JMP, version 8.0 (SAS Institute) and SAS, version 9.2 (SAS Institute). Continuous variable distributions were checked for normality. Where satisfactory, continuous data were summarized as mean (SD); where skewed, median (interquartile range [IQR]) was reported. Discrete variables were expressed as frequencies with percentages. For the primary analysis comparing cases (with recurrence) and controls (without recurrence), conditional logistic regression was used to assess for variable differences while accounting for matching. The Kaplan-Meier curve was used to estimate recurrent SCAD among all women at risk. As a secondary analysis, a Cox proportional hazards model of time to first SCAD recurrence was fit using subsequent (post-SCAD) pregnancy as a time-dependent factor, adjusted for the time-independent covariates of age at first SCAD, year of first SCAD, and FMD status. For all time-to-recurrence analyses, patients were censored at last follow-up or the date that they turned 56 years of age, whichever came first. Missing data was remediated by chart review, patient contact, or mentioned as a table footnote. A 2-sided *P* < .05 was considered statistically significant.

## Results

### Case Series of 23 Women, 32 Pregnancies

Within the overall cohort, 23 women (median [IQR] age, 38 [34-40] years; 20 White [87%]) were identified as having a total of 32 post-SCAD pregnancies. Most were older mothers with a median (IQR) time to conception after SCAD of 18 (12-23) months (Table 1). Seven women (30%) had a history of SCAD within 12 weeks postpartum, 9 (39%) had a history of migraines, and 6 (26%) had a history of FMD. Five women (22%) had more than 1 post-SCAD pregnancy. Eleven of the 32 pregnancies (34%) were miscarriages; 1 woman had 6 miscarriages (2 post-SCAD). Twenty pregnancies (63%) resulted in live births including 13 term vaginal deliveries (65%), 6 term cesarean deliveries (30%), and 1 emergent preterm cesarean section (5%). Other than the preterm delivery, concerns were limited to 5 women with (1) mild hypertension, (2) benign arrhythmia, (3) severe vertigo, (4) nonspecific spells with normal evaluation, and (5) postpartum hemorrhage. Fifteen women (75%) breastfed their infants for a median (IQR) of 4.8 (2-36) months, and 1 was still breastfeeding at time of follow-up. Two discontinued breastfeeding, 1 owing to chest pain that occurred with breastfeeding and the other owing to recurrent SCAD.

**Table 1.** Baseline Characteristics of Women With Pregnancy After SCAD

Patient characteristic	No. (%)^a^
Age at time of post-SCAD pregnancy, median (IQR) [range], y	38 (34-40) [28-42]
Time from SCAD to pregnancy, median (IQR), mo	18 (12-23)
Year of 1st SCAD, median (IQR) [range]	2012 (2009-2014) [1992-2017]
White	20 (87)
BMI, mean (SD), kg/m^2^	25 (6)
Gravida, median (IQR) [range]	3 (2-4) [1-8]
Parity, median (IQR) [range]	2 (2-4) [0-7]
No. of pregnancies after SCAD, median (range)	1 (1-4)
FMD	
No	5 (22)
Yes	6 (26)
Non-FMD extracoronary vascular abnormality	1 (4)
Unknown or not imaged	11 (48)
Migraines	9 (39)
Hyperlipidemia	4 (17)
Hypertension	6 (26)
Connective tissue disease	2 (9)
Diabetes	1 (4)
Hypothyroidism	2 (9)
History of infertility therapy	3 (13)
Polycystic ovarian syndrome	1 (4)
Multiple sclerosis	1 (4)
Tobacco use	
Prior	3 (13)
Active	1 (4)
Details of initial SCAD	
Age at SCAD, mean (SD), y	35 (4)
SCAD	
During pregnancy, n	0
While postpartum (≤12 wk)	7 (30)
While postpartum up to 13 mo	11 (48)
Associated with recent	
Extreme exertion	2 (9)
Extreme stress/emotion	9 (40)
Myocardial infarction	
ST segment elevation	8 (35)
Non-ST segment elevation	14 (61)
During presentation	
Ventricular fibrillation	2 (9)
Ventricular tachycardia	2 (9)
Unstable angina	1 (4)
Multivessel SCAD	6 (26)
Coronary territory affected by SCAD	
Left main	2 (9)
Left anterior descending	13 (57)
Ramus	2 (9)
Diagonal	2 (9)
Left circumflex	4 (17)
Obtuse marginal	5 (22)
Right coronary artery	4 (17)
Posterior descending artery	1 (4)
Posterolateral artery	0
Medical management only	13 (57)
Progression	1 of 13 (8)
PCI	9 (39)
Successful	8 of 9 (89)
Complicated	2 of 9 (22)
Coronary artery bypass grafting	2 (9)
Left ventricular ejection fraction, median (IQR) [range], %	55 (44-60) [25-65]^b^
Outcomes of 32 pregnancies	
1st trimester	
Miscarriage	9 (28)
Elective termination	1 (3)
2nd trimester miscarriage	2 (6)
Preterm delivery	1 (3)
Term delivery	19 (59)
Details of 20 deliveries	
Vaginal delivery	
Spontaneous	5 (25)
Induced	7 (35)
Cesarean section	
Intrapartum	1 (5)
Elective	6 (30)
Emergency	1 (5)
Pain management strategy of 20 deliveries	
Epidural	13 (65)
Spinal	4 (20)
General anesthesia	1 (5)
Nitrous oxide	1 (5)
None	1 (5)
Breastfeeding	
Breastfeeding after 20 deliveries	15 (75)
Length of breastfeeding, median (range), mo	4.8 (2-36)
Contraception after pregnancy among 23 women^c^	
None	7 (30)
Partner sterilization	5 (22)
Intrauterine device	4 (17)
Tubal ligation	3 (13)
Condoms	2 (9)
Hysterectomy	1 (4)
Endometrial ablation	1 (4)
Minipill	1 (4)

Abbreviations: BMI, body mass index; FMD, fibromuscular dysplasia; IQR, interquartile range; PCI, percutaneous coronary intervention; SCAD, spontaneous coronary artery dissection.

^a^ Values are expressed as No. (%) unless otherwise specified.

^b^ All patients with a left ventricular ejection fraction <50% at the time of initial SCAD had improvement in subsequent function to ≥54% before pregnancy after SCAD including 3 patients with initial left ventricular ejection fraction measurements of <35%.

^c^ Some patients used multiple contraception methods.

Among the 23 women who had any post-SCAD pregnancy, the median (IQR) time from initial SCAD to first post-SCAD pregnancy was 1.5 (1-1.9) years. The median (IQR) length of follow-up from initial SCAD was 7.1 (4.5-10.0) years, and the median (IQR) time from most recent pregnancy to last follow-up was 4.3 (2.4-8.9) years. Two of these women (9%) experienced recurrent SCAD. The first had initial SCAD 16 months following the birth of her third child and life-threatening recurrent SCAD at 9 weeks postpartum as described previously.^26^ At 8 years postpregnancy, she reported intermittent severe fatigue but no major interim medical events. The second woman with recurrence had extensive FMD and her first SCAD 12 weeks postpartum after a complicated vaginal delivery of her second child. She then had 3 additional pregnancies, the last of which was a miscarriage. Her 2 recurrent SCADs occurred 12 and 21 years after her final pregnancy.

### Nested Case-Control Analysis of Patients With Recurrent SCAD

Ninety-two women with recurrent SCAD were matched to 158 controls (Table 2). Five women with recurrence could not be matched. Among women with SCAD recurrence, 26 had 1 match and 66 had 2 matches. Overall, there was no significant difference in history of subsequent pregnancies in the women with recurrent SCAD as compared to matched controls, with some evidence of more subsequent pregnancies in the control group (2 of 92 [2%] vs 13 of 158 [8%]; *P* = .06). Both groups had similar numbers of prior pregnancies.

**Table 2.** Baseline Data for Patients in the Nested Case-Control Analysis Including the Total Number at Risk

Patient characteristic	No. (%)^a^			*P* value
	At risk (n = 636)	Cases (n = 92)	Matched controls (n = 158)	
Gravida, median (IQR) [range]	2 (2-4) [0-10]	2 (2-4) [0-10]	2 (2-4) [0-9]	.54
Parity, median (IQR) [range]	2 (2-3) [0-7]	2 (2-3) [0-7]	2 (1-3) [0-7]	.03
Subsequent pregnancy	23 (3.6)	2 (2.2)	13 (8.2)	.06
No. of pregnancies after SCAD, No.				
1	18	1	11	NA
2	2	0	2	
3	2	1	0	
4	1	0	0	
Time from SCAD to 1st pregnancy, median (IQR) [range], mo	17.8 (12-23) [2.8-39]	3.9 (2.8-4.9) [2.8-4.9]	19 (13-23) [3.6-39]	NA
Age at time of 1st post-SCAD pregnancy, median (IQR) [range], y	38 (34-40) [28-42]	33.5 (33-34) [33-34]	38 (35-40) [34-42]	NA
White	586 (92)	84 (91)	147 (93)	.61
BMI, mean (SD) [range], kg/m^2^	26.6 (6.5) [15.2-59.5]	26.5 (6.4) [15.2-49.5]	25.7 (6.3) [17.3-59.5]	.22
FMD				
No	144 (22.6)	15 (16.3)	30 (19.0)	NA
Yes	232 (36.5)	45 (48.9)	71 (44.9)	
Possible	29 (4.6)	7 (7.6)	12 (7.6)	
Non-FMD extracoronary vascular abnormality	41 (6.4)	7 (7.6)	11 (7.0)	
Unknown or not screened	190 (29.9)	18 (19.6)	34 (21.5)	
Migraines	232 (36.5)	36 (39.1)	63 (39.9)	.70
Hyperlipidemia	155 (24.4)	34 (36.9)	31 (19.6)	.007
Hypertension	164 (25.7)	30 (32.6)	47 (29.7)	.69
Connective tissue disease	12 (1.9)	5 (5.4)	2 (1.3)	.11
Diabetes	20 (3.1)	3 (3.3)	4 (2.5)	.67
Hypothyroidism	75 (11.8)	18 (19.6)	18 (11.4)	.08
History of infertility therapy	78 (12.3)	14 (15.2)	19 (12.0)	.40
Polycystic ovarian syndrome	19 (3.0)	1 (1.1)	6 (3.8)	.28
Prior tobacco use	156 (24.5)	18 (19.6)	37 (23.4)	.48
Active tobacco use	15 (2.4)	1 (1.1)	6 (3.8)	.31
Age at 1st SCAD, mean (SD) [range], y	41.7 (6.7) [17.4-55.8]	40.9 (6.0) [28.0-54.9]	40.8 (5.5) [25.3-53.3]	.27
Year of 1st SCAD, median (IQR) [range]	2013 (2011-2016) [1986-2019]	2010 (2007-2012) [1991-2017]	2012 (2009-2014) [1989-2018]	<.001
SCAD				
During pregnancy	7 (1.1)	1 (1.1)	2 (1.3)	.84
While postpartum (≤12 wk)	84 (13.2)	8 (8.7)	22 (13.9)	.14
While postpartum up to 13 mo	113 (17.8)	16 (17.4)	32 (20.3)	.42
Associated with recent				
Extreme exertion	97 (15.3)	17 (18.5)	25 (15.8)	.63
Extreme stress/emotion	134 (21.1)	18 (19.6)	29 (18.4)	.82
Myocardial infarction^b^				
ST segment elevation	259 (40.7)	42 (45.7)	62 (39.2)	.34
Non-ST segment elevation	358 (56.3)	48 (52.1)	91 (57.6)	.47
During presentation				
Ventricular fibrillation	62 (9.7)	3 (3.3)	21 (13.3)	.02
Ventricular tachycardia	58 (9.1)	7 (7.6)	12 (7.6)	.73
Unstable angina	14 (2.2)	1 (1.1)	2 (1.3)	.70
Multivessel SCAD	133 (21.0)	17 (18.5)	36 (22.8)	.28
Coronary territory affected by SCAD				
Left main	59 (9.3)	6 (6.5)	14 (8.9)	.62
Left anterior descending	396 (62.3)	49 (53.3)	107 (67.7)	.02
Ramus	21 (3.3)	2 (2.2)	5 (3.2)	.60
Diagonal	49 (7.7)	4 (4.3)	10 (6.3)	.39
Left circumflex	90 (14.2)	18 (19.6)	23 (14.6)	.29
Obtuse marginal	142 (22.3)	27 (29.3)	27 (17.1)	.02
Right coronary artery	70 (11.0)	13 (14.1)	16 (10.1)	.37
Posterior descending artery	55 (8.6)	8 (8.7)	15 (9.5)	.82
Posterolateral artery	21 (3.3)	6 (6.5)	2 (1.3)	.046
Medical management only	339 (53.3)	43 (46.7)	71 (44.9)	.75
Progression among those with medical management	47 (13.9)	2 (4.7)	14 (19.7)	NA
PCI	261 (41.0)	45 (48.9)	77 (48.7)	.89
Successful	208 (79.7)	32 (71.1)	61 (79.2)	.21
Complicated	83 (31.8)	17 (37.8)	25 (32.5)	.22
Coronary artery bypass grafting	56 (8.8)	9 (9.8)	19 (12.0)	.51
Left ventricular ejection fraction, mean (SD) [range], %^c^	51.9 (11.8) [15-77]	53.1 (10.5) [20-75]	51.5 (11.1) [20-77]	.41
Follow-up time, median (IQR) [range], y				
After 1st SCAD	3.2 (1.4-5.7) [0.09-31.0]	3.4 (1.4-6.3) [0.09-17.9]	5.24 (3.5-8.7) [0.32-28.9]	<.001
After most recent pregnancy	4.3 (2.4-8.9) [0.22-22.7]	15.8 (8.8-22.7) [8.8-22.7]	6.4 (2.4-9.6) [0.22-12.8]	NA

Abbreviations: BMI, body mass index; FMD, fibromuscular dysplasia; IQR, interquartile range; PCI, percutaneous coronary intervention; SCAD, spontaneous coronary artery dissection.

^a^ Values are expressed as No. (%) unless otherwise specified.

^b^ Five patients did not have information to clearly define the subtype of acute coronary syndrome.

^c^ Sixty-one patients did not have acute left ventricular ejection fraction data available.

### Cohort Time to Event

Of the 636 women of childbearing potential in the SCAD cohort, 23 (3.6%) had a pregnancy after SCAD. If recurrent SCAD was defined as occurring any time after the initial SCAD event, 122 of 636 of the cohort (19%) had a recurrence (18.6% cumulative incidence at 5 years’ follow-up by Kaplan-Meier analysis; 95% CI, 14.6%-22.4%). If recurrent SCAD was restricted to occurring at least 1 month after the initial SCAD, 97 of 636 of the cohort (15%) had a recurrence (14.8% at 5 years of follow-up by Kaplan-Meier analysis; 95% CI, 11.1%-18.5%). The Cox analysis when controlling for age at first SCAD, year of first SCAD, and FMD status showed no significant association between subsequent pregnancy and SCAD recurrence with nonsignificant hazard ratios of 0.38 (95% CI, 0.09-1.6; *P* = .19) and 0.44 (95% CI, 0.10-1.82; *P* = .26) for both definitions of recurrence (Table 3 and the eTable in the Supplement). Those patients with an unknown evaluation for FMD had significantly fewer recurrences with hazard ratios of 0.41 (95% CI, 0.22-0.77; *P* = .005) and 0.39 (95% CI, 0.19-0.81; *P* = .01), respectively. Two of the 636 women (0.3%) who did not have pregnancy after SCAD died during follow-up. One death was related to recurrent SCAD and cardiac arrest not associated with pregnancy; the other death was due to cancer. There are no reports of sudden deaths caused by SCAD and pregnancy in follow-up of the 636 participants.

**Table 3.** Univariable and Multivariable Cox Analysis of SCAD Recurrence and Pregnancy After SCAD With Recurrence Timeline of at Least 1 Month After Initial SCAD

Variable	Univariable analysis		Multivariable analysis	
	Unadjusted HR (95% CI)	*P* value	Adjusted HR (95% CI)	*P* value
Age at 1st SCAD	0.99 (0.96-1.02)	.44	0.98 (0.95-1.01)	.28
Year of 1st SCAD	1.03 (0.99-1.07)	.19	1.02 (0.98-1.07)	.33
Time-dependent subsequent pregnancy^a^	0.35 (0.09-1.42)	.14	0.38 (0.09-1.60)	.19
FMD^b^	1.21 (0.73-1.99)	.46	1.29 (0.77-2.14)	.33
Possible FMD	1.09 (0.46-2.56)	.84	1.20 (0.51-2.87)	.68
Non-FMD EVA	1.52 (0.72-3.21)	.27	1.55 (0.73-3.27)	.26
Unknown or not screened for FMD	0.37 (0.20-0.68)	.002	0.41 (0.22-0.77)	.005

Abbreviations: EVA, extracoronary vascular abnormality; FMD, fibromuscular dysplasia; HR, hazard ratio; SCAD, spontaneous coronary artery dissection.

^a^ The model accounted for time to each individual’s subsequent pregnancy.

^b^ No FMD was used as the baseline level.

## Discussion

These findings suggest that SCAD is multifactorial with contributory factors beyond pregnancy alone. SCAD recurrence was uncommon in the case series of women with pregnancy after SCAD. Within the nested case-control analysis, women with SCAD recurrence were less likely to have a history of subsequent pregnancy compared with those without SCAD recurrence. In the cohort analysis, a history of subsequent pregnancy was not associated with SCAD recurrence. This unexpected finding may be in part because of the small number of women who had another pregnancy after SCAD. Although the small number of women notably limits the power of this analysis, it also reflects the fact that most women do not become pregnant after SCAD, consistent with current recommendations. However, these data also highlight the complexity of SCAD. It is likely not a single precipitator (eg, pregnancy, stress, exercise) that leads to SCAD onset, but most likely a complex combination of several appreciated and unappreciated factors.

It is reassuring that the majority of women tolerated pregnancy and lactation after SCAD without substantial complications. Given the potential severe consequences of a recurrent SCAD for which there are not yet preventive strategies, our findings should be interpreted with caution. This study does not constitute sufficient evidence to revise current recommendations to avoid pregnancy after SCAD (Box) with preferred contraceptive options being partner sterilization, tubal ligation, or intrauterine devices.^1^ The pregnant state represents a cardiovascular “stress test” with physiologic increases in blood volume, heart rate, and cardiac output that may especially affect women with residual cardiac dysfunction, arrhythmias, or those requiring specific medications. Should SCAD recur, pregnancy-associated SCAD has been observed as having a severe presentation.^1,2,27^

Box. **Summary of Clinical Considerations**

**Rationale for Recommending Against Pregnancy After SCAD**

- SCAD is associated with the pregnant and postpartum status
- Pregnancy-associated SCAD is often severe and life-threatening
- Following an initial SCAD episode, patients may have baseline left ventricular dysfunction, arrhythmias, and require cardiovascular medications
- Predictors for recurrent SCAD are unknown
- Preventive therapies for recurrent SCAD are unknown

**Approach to a Patient Contemplating Pregnancy (or Already Pregnant)**

- Multidisciplinary Pregnancy Heart Team approach including specialists in Cardiology, Anesthesiology, and Maternal-Fetal Medicine with individualized risk assessment, counseling, and care plan
- Confirm details of SCAD history and treatment
- Evaluate current cardiac function, symptoms, and medications
- Review prior imaging for arteriopathy such as FMD and update as appropriate
- Mode and timing of delivery per standard obstetric indications with a preference for:
  - Level IV maternal care facility capable of managing cardiac emergencies
  - Planned vaginal delivery and neuraxial anesthesia

Abbreviations: FMD, fibromuscular dysplasia; SCAD, spontaneous coronary artery dissection.

The fundamental desire for biological children can be paramount for some women, and they may proceed with planned or unplanned pregnancy despite these recommendations. Our findings indicate that not all women with pregnancy after a SCAD MI are ultimately destined to have another SCAD. Rather, an unremarkable clinical course was most often observed. If a woman strongly desires pregnancy or continues with an unintended pregnancy after SCAD, counseling is critical with input from a multidisciplinary “pregnancy heart team” comprised of specialists in cardiology, maternal-fetal medicine, and anesthesiology. Recommendations should be guided by current consensus statements and risk stratifications, thereby tailoring advice to the patient’s personal cardiac history and medication regimen.^1,2,28^ Considerations specific to SCAD include knowing the presence of FMD and other arterial abnormalities such as aneurysms in other arteries, understanding the current cardiac function, and reviewing any ongoing symptomology. An important caveat is that the women in this series who presented with a reduction in left ventricular ejection fraction at the time of SCAD recovered to a normal or near-normal ejection fraction before pregnancy. Outcomes may not be as reassuring among women with persistent left ventricular systolic dysfunction or other active concerns, such as chronic angina. For instance, a left ventricular ejection fraction less than 30% is associated with a high risk of morbidity and mortality and considered a contraindication to pregnancy according to the modified World Health Organization criteria, regardless of origin.^28,29^

### Limitations

Limitations of this study include the small number of women with pregnancy after SCAD. This study is based on participants of a registry cohort; therefore, selection, referral, and recall bias may confound the findings. Particularly as the success of the Mayo Clinic Registry is in part due to social networking, the younger mean age and higher prevalence of pregnancy-associated SCAD in this cohort as compared with the Canadian cohort^8^ may be a reflection of biases inherent to the registry. Those who had an unknown history or were not screened for FMD had significantly less recurrence in the Cox proportional analysis. This may reflect that those with recurrence may be more likely to be aggressively evaluated for an etiology such as FMD. It is also possible that those who are less likely to seek or report evaluation for FMD may be less likely to seek or report evaluation for symptoms of another SCAD. Finally, all of the women who became pregnant after SCAD had normal or near-normal cardiac function at the onset of pregnancy, which is not generalizable to all women with history of SCAD.

## Conclusions

This study found no association of SCAD recurrence with pregnancy among women with prior SCAD. Most women had minimal to no complications with pregnancy and lactation after SCAD, which is reassuring and suggests that multiple factors contribute to the onset of SCAD. However, these findings must be interpreted cautiously because of the study limitations. Regardless, because of the rarity of this condition, prospective trials are unlikely, and counseling will be predicated on data such as these despite inherent limitations. Further elucidation of genetic predisposition and underlying mechanisms may eventually allow some degree of recurrence prediction. Until then, optimal care requires considerate counseling and collective decision-making.

## Notes

Supplement. **eTable.** Univariable and Multivariable Cox Analysis Showed No Significant Correlation With SCAD Recurrence and Pregnancy After SCAD When the SCAD Timeline is Defined as Occurring Any Time After the Initial SCAD
