# Supplementary material for: Association of Pregnancy With Recurrence of Spontaneous Coronary Artery Dissection Among Women With Prior Coronary Artery Dissection
Source: JAMA Netw Open. 2020 Sep 23;3(9):e2018170. doi: 10.1001/jamanetworkopen.2020.18170 (PMC7512056; doi:10.1001/jamanetworkopen.2020.18170)
Supplement: Supplement. — eTable. Univariable and Multivariable Cox Analysis Showed No Significant Correlation With SCAD Recurrence and Pregnancy After SCAD When the SCAD Timeline is Defined as Occurring Any Time After the Initial SCAD [file jamanetwopen-e2018170-s001.pdf]

Supplementary Online Content

Tweet MS, Young KA, Best PJM, et al. Association of pregnancy with recurrence of spontaneous coronary artery dissection among women with prior coronary artery dissection. *JAMA Netw Open*. 2020;3(9):e2018170. doi:10.1001/jamanetworkopen.2020.18170

eTable. Univariable and Multivariable Cox Analysis Showed No Significant Correlation With SCAD Recurrence and Pregnancy After SCAD When the SCAD Timeline is Defined as Occurring Any Time After the Initial SCAD

This supplementary material has been provided by the authors to give readers additional information about their work.

© 2020 Tweet MS et al. *JAMA Network Open.*

eTable. Univariable and Multivariable Cox Analysis Showed No Significant Correlation With SCAD Recurrence and Pregnancy After SCAD When the SCAD Timeline is Defined as Occurring Any Time After the Initial SCAD

	Univariable analysis			*Multivariable analysis*		
Variable	*Unadjusted HR*	*95% CI*	*p value*	*Adjusted HR*	*95% CI*	*p value*
Age at 1st SCAD	1.00	(0.96, 1.03)	0.89	0.99	(0.96, 1.03)	0.59
Year of 1st SCAD	1.03	(0.98, 1.08)	0.25	1.03	(0.98, 1.07)	0.31
Time-dependent Subsequent Pregnancy†	0.36	(0.09, 1.49)	0.16	0.44	(0.10, 1.82)	0.26
FMD*	1.37	(0.76, 2.47)	0.30	1.45	(0.80, 2.64)	0.22
Possible FMD	1.51	(0.61, 3.72)	0.37	1.66	(0.66, 4.16)	0.28
Non-FMD EVA	1.32	(0.54, 3.25)	0.54	1.40	(0.57, 3.47)	0.47
Unknown or not screened for FMD	0.35	(0.17, 0.72)	0.005	0.39	(0.19, 0.81)	0.012

*No FMD was used as the baseline level
†The model accounted for time to each individual's subsequent pregnancy.

CI=confidence interval; EVA=extracoronary vascular abnormality; FMD=fibromuscular dysplasia; HR=hazard ratio; PCI=percutaneous coronary intervention; SCAD=spontaneous coronary artery dissection

© 2020 Tweet MS et al. *JAMA Network Open.*
